# 

**Published:** 1870-10

**Authors:** 


					﻿VOL. XXIV.	No. 10.
THE
Dental Register.
EDITED BY
J. TAFT & Gr. WATT.
OCTOBER, 1870.
MONTHLY:
THREE DOLLARS PER ANNUM, IN ADVANCE.
CINCINNATI:
WRIGHTSON & CO., Printers, 167 WALNUT STREET.
J. A WTtT.	Dmti.it.i, 117 West f'mirtli St.
				

